# Investigating the Relevance of Genetic Variation at Post‐Translational Modification Sites in the Polygenic Risk Model for Alzheimer’s disease‐associated Phenotypes

**DOI:** 10.1002/alz.090825

**Published:** 2025-01-09

**Authors:** Samantha LG Paço, Felipe Luiz Pereira, Shea J Andrews, Lea T. Grinberg, Michel Satya Naslavsky

**Affiliations:** ^1^ Universidade de São Paulo, São Paulo, Sao Paulo Brazil; ^2^ Weill Institute for Neurosciences and Memory and Aging Center, Department of Neurology, University of California, SF, CA USA; ^3^ University of California, San Francisco, San Francisco, CA USA; ^4^ Department of Neurology, University of California at San Francisco, San Francisco, CA USA; ^5^ Memory & Aging Center, Department of Neurology, University of California in San Francisco, San Francisco, CA USA; ^6^ Biobank for aging studies of the University of São Paulo Medical School, Brazil, São Paulo Brazil; ^7^ University of São Paulo, São Paulo Brazil

## Abstract

**Background:**

The outcomes of extensive Genome‐Wide Association Study (GWAS) data in Polygenic Risk Score (PRS) studies exhibit varying odds ratios (ORs), ranging from 1.13 (Bellenguez) to 1.34 (Kunkle). These diverse outcomes stem from inherent limitations within the tool, including SNP selection criteria, rare variant exclusion, the absence of biological information integration, and considerations for diverse populations.

Overcoming PRS limitations and enhancing efficiency requires strategic integration of genetic loci annotations with post‐translational modifications (PTMs), impacting AD‐related processes and genes (NFT, PSEN1, PSEN2, APP).

Furthermore, the inclusion of diverse populations, specifically working with admixed populations like the Brazilian dataset, is essential for a nuanced understanding of these factors. This approach is critical for a comprehensive application of PRS in advancing our understanding of Alzheimer’s disease.

Consequently, The main goal is to evaluate PTM impact on Alzheimer’s disease phenotypes using Polygenic Risk Score models in Brazilian and European populations.

**Method:**

The initial outcomes of the project involve the establishment of the PTMflex Annotator. This annotator seamlessly integrates the PTMnet and PhosphoSitePlus datasets with functional protein information from the NCBI platform. Serving as a unified tool, it facilitates efficient access and exploration of PTM sites and types. Users can input parameters such as genomic position, Ref seq transcript, or protein ID for precise inquiry.

The whole‐genome sequence datasets from the European Population (ADNI) and the Brazilian Population (ABraOM) will be integrated into the PTMflex Annotator for PTM extraction and verification. Subsequent tasks include logistic regression to determine PTM weights for each variant and calculating the total PTMs feature of each sample. The project concludes with a comprehensive PRS enrichment analysis, assessing the combined impact of PTMs and the PRS model through PLINK software, utilizing PGS003957 ‐ Kunkle (12,002 Variants) and PGS003953 ‐ Bellenguez (1,937 Variants) as base dataset.

**Result:**

The ongoing phases of the project include applying target data into the PTMflex Annotator.

**Conclusion:**

Anticipating discernible differences in the influence of PTMs within the prediction model across the two populations, the comprehensive whole‐genome sequence dataset is expected to identify rare variants linked to PTMs that could impact the Alzheimer’s disease phenotype.

